# Prevalence and Costs of Multimorbidity by Deprivation Levels in the Basque Country: A Population Based Study Using Health Administrative Databases

**DOI:** 10.1371/journal.pone.0089787

**Published:** 2014-02-27

**Authors:** Juan F. Orueta, Arturo García-Álvarez, Manuel García-Goñi, Francesco Paolucci, Roberto Nuño-Solinís

**Affiliations:** 1 Centro de Salud de Astrabudua, Osakidetza - Basque Health Service, Erandio, Bizkaia, Spain; 2 O+Berri, Basque Institute for Healthcare Innovation, Sondika, Bizkaia, Spain; 3 Departamento de Economía Aplicada II, Universidad Complutense de Madrid, Madrid, Spain; 4 The Australian National University, Acton, Australia; 5 University of Northumbria, Newcastle upon Tyne, United Kingdom; CUNY, United States of America

## Abstract

**Background:**

Multimorbidity is a major challenge for healthcare systems. However, currently, its magnitude and impact in healthcare expenditures is still mostly unknown.

**Objective:**

To present an overview of the prevalence and costs of multimorbidity by socioeconomic levels in the whole Basque population.

**Methods:**

We develop a cross-sectional analysis that includes all the inhabitants of the Basque Country (N = 2,262,698). We utilize data from primary health care electronic medical records, hospital admissions, and outpatient care databases, corresponding to a 4 year period. Multimorbidity was defined as the presence of two or more chronic diseases out of a list of 52 of the most important and common chronic conditions given in the literature. We also use socioeconomic and demographic variables such as age, sex, individual healthcare cost, and deprivation level. Predicted adjusted costs were obtained by log-gamma regression models.

**Results:**

Multimorbidity of chronic diseases was found among 23.61% of the total Basque population and among 66.13% of those older than 65 years. Multimorbid patients account for 63.55% of total healthcare expenditures. Prevalence of multimorbidity is higher in the most deprived areas for all age and sex groups. The annual cost of healthcare per patient generated for any chronic disease depends on the number of coexisting comorbidities, and varies from 637 € for the first pathology in average to 1,657 € for the ninth one.

**Conclusion:**

Multimorbidity is very common for the Basque population and its prevalence rises in age, and unfavourable socioeconomic environment. The costs of care for chronic patients with several conditions cannot be described as the sum of their individual pathologies in average. They usually increase dramatically according to the number of comorbidities. Given the ageing population, multimorbidity and its consequences should be taken into account in healthcare policy, the organization of care and medical research.

## Introduction

Most OECD countries face major challenges in (re-)organising the funding and provision of care to respond to the increasing demands of patients with chronic diseases. Notably, most of those patients suffer more than one chronic condition at the same time and become multimorbid patients [1]. In fact, it has been suggested that multimorbidity itself is the “most prevalent chronic condition” [2],[3]. Individuals manifesting multimorbidities typically have a lower quality of life, and higher degree of disability, psychological distress, risk of mortality, and utilisation of health (and social) services than if we considered their chronic conditions in isolation or individuals with a single chronic condition [4]. Therefore, it is widely accepted that health systems need to focus their strategies for confronting such problem.

Although most studies on this topic refer to the re-organisation of provision [5], [6], the economic impact is a major concern given the concentration of health expenditures on these patients [7]. This health policy challenge is particularly important in countries where there is a significant proportion of public funding, and even more so in regions affected by the current poor economic situation and outlook, including Spain. In these countries, public policy makers find themselves involved in an urgent search for efficiency in provision while still guaranteeing quality with equity in access, in many systems in the context of universal coverage.

The relationship between individual healthcare expenditures and demographic characteristics and/or morbidity has been extensively explored [8]. Nevertheless, the real impact that individuals with multimorbidity have (and will have in the near future) on healthcare costs and on the organization and management of healthcare provision and financing is yet to be seen and only recently a few authors have addressed this topic from an empirical perspective [9]. Among those who have, some have found a nearly linear relationship between increases in the number of chronic conditions and individual's healthcare costs [10], [11], while others provide evidence of an exponential rise in costs for patients with multimorbidity [12]. In this paper, we aim to contribute to this niche in the literature [4], [13].

We exploit a unique database from the Basque Country, a region in Spain, to look together at multimorbidity, healthcare expenditures, and socioeconomic characteristics. In this way, we present an analysis of the state of the art in the Basque Country with respect to its health provision and planning, describe health expenditures, and identify the real role and importance of multimorbidity in the concentration of healthcare costs. Given the similarities between the Basque population and the society and demographics of most economically developed countries, our insights into what we have learned in the Basque Country may be extrapolated to other populations.

The objectives of this study are, first, to describe the map of prevalence of chronic conditions and multimorbidity in the Basque population; second, to observe that prevalence in socioeconomic groups defined by a deprivation index [DI]; third, to present the level of health expenditures on different types of healthcare provision by individuals controlling for the presence of multimorbidity and deprivation.

## Methods

This study utilized the database prepared by the population stratification programme (PREST) of the Basque Country. Such database is property of the Basque Health Service and the access to it is restricted. The study population included every individual covered on 31 August 2011 by the public health insurance in the Basque Country and who had been covered for at least 6 months in the previous year, regardless of whether they had made any contact with or use of the Basque Health Service or not. That is, practically all the inhabitants of the Basque Country are included. Diagnoses on hospital discharge forms, emergency department databases and primary care medical records are coded according to ICD-9-CM [14], while the ATC [15] coding system is used for drugs prescribed by primary care doctors. With this information, residents in the Basque Country are classified annually using ACGs [16]. Demographic variables including age on the final day of the study period, and gender, were collected, along with the area-based DI, chronic diseases recorded and yearly health costs.

The total population considered in the study is 2,262,707, of which 50.90% are female. As for the age distribution, 15% of patients are children (age <18) and 20% are over 65.

### Ethical Statement

The Clinical Research Ethics Committee of the Basque Country approved this study according to the Spanish Law 14/2007 on Biomedical Research, the Ethical Principles for Medical Research of the Declaration of Helsinki and other applicable ethical principles. We used databases that employ an opaque identifier to ensure patient confidentiality. Written consent by the patients was specifically waived by the approving Committee.

### Setting

The Basque Country is one of the Spanish autonomous regions with longest experience (since 1983) in managing competences in health planning and provision and a pioneer in the transition towards a chronic care model in Spain [17], [18]. In 2010 the Department of Health and Consumer Affairs of the Autonomous Region of the Basque Country launched its “Strategy for tackling the challenge of chronicity in the Basque Country” [19]. This strategy outlines the key guiding principles to improve the responsiveness of the Basque healthcare system to chronic patients' demands and thereby to enhance its efficiency and long-term financial sustainability, as well as the quality of care for chronic patients.

An important component of the strategy is to develop a tool for the risk stratification of the entire population of the Basque Country. For this purpose, a large dataset was assembled combining information on individuals from several sources (namely, primary and specialized health care records, and census data) and including clinical - and utilisation data (e.g., diagnoses, prescriptions, categorization of people according to their disease burden by means of the Adjusted Clinical Groups case-mix system [ACG] [16], [20], healthcare costs and an area-based DI).

The Basque Health System (Osakidetza) is an organization publicly funded through general taxes. It provides universal coverage to the citizens in the Basque Country. At the point of delivery, provision of care is free of charge, with the exception of pharmaceuticals, which can entail a co-payment. The percentage charged varies according to the type of illness, the level of income of the patient and his/her age.

### Chronic Conditions and the Compilation of Data

Our study draws on various sources of information, and this helps to overcome some of the shortcomings of using any of them separately. In organizations such ours, where every resident is registered on the list of a GP who acts as a gatekeeper for other levels of care, primary care records are considered a reliable guide to the prevalence of chronic illnesses. However, the extent to which the records of diagnoses are complete is influenced by a range of factors [21],[22],[23] and their combined use with inpatient and outpatient specialised care can produce more accurate estimates [24]. On the other hand, prescriptions records can provide adequate information to identify patients with some conditions [25], [26]. However, an exhaustive process of data capture may lead to overestimation, detecting illnesses that are long-lasting, but not currently active. In a recent study, Barnett et al. (2012) developed a mixed method to identify 40 chronic diseases [27], establishing specific criteria for each; depending on the characteristics of the pathology, in some cases an isolated diagnosis is accepted, in others prescriptions, diagnosis plus prescriptions in the last year or a given number of prescriptions are required.

In the present study, we considered four years of data for each of the individuals in the Basque Country. Taking advantage of and adapting the aforementioned methodology [27], we developed a list of 52 health conditions and defined a specific criterion for each to consider it active. A description of this process is included as supporting material (File S1). For the purposes of the study, multimorbidity was defined as the coexistence of two or more of these conditions in the same patient.

### Healthcare Costs

For the period between 1 September 2010 and 31 August 2011, we estimated healthcare costs of primary care prescriptions recorded in electronic health records based on the market value of the drugs. For other variables (e.g., visits to Accident & Emergency [A&E], rehabilitation sessions, outpatient care, primary care visits, laboratory tests and radiological examinations ordered by primary care, and various outpatient procedures such as dialysis, radiotherapy and chemotherapy), the number of services used by each patient was multiplied by standardized costs (the average cost of each service provided to a patient treated in Osakidetza, according to calculations made by the aforementioned organisation). The costs of hospitalisation and outpatient surgery were calculated in relation to their cost-weights in the corresponding diagnosis-related groups (DRGs). Some services for which it was not possible to obtain information were excluded; these include admission to psychiatric hospitals, home care and day care services (except for the procedures mentioned above), transportation, and prostheses and other equipment provided to patients at home.

### Socioeconomic Information

The DI, defined by census tract, was used as a socioeconomic indicator. This index is an ordinal variable, categorized into five levels (DI quintiles), providing a measure of the socioeconomic characteristics of the population of census tracts. The DI was elaborated and published in 2008. Its design allows for the estimation of socioeconomic and environmental inequalities among inhabitants by censal code in Spain. Its calculus takes into account the percentages of residents who are manual workers, unemployed, temporary employees, or have an inadequate level of educational attainment, overall and also specifically among young people [28], given the most recent Census (2001) available.

### Statistical Analysis

Tables 1–7 present the descriptive statistics of our data. In order to compare healthcare cost for individuals belonging to the different categories of the DI given their number of chronic conditions, we utilize the Kruskal-Wallis non-parametric test.

**Table 1:** Averages of chronic diseases and percentage of patients with comorbidity in patient groups according to sex, age and deprivation index.

		Average number of chronic illnesses per patient						Number and percentage (%) of patients with multimorbidity (2+ chronic diseases)					
		General average	Deprivation index					Deprivation index					All
	No. People		1	2	3	4	5	1	2	3	4	5	
ALL	2,262,698	0.97	0.84	0.92	0.98	1.06	1.08	98,356 (20.52%)	108,992 (22.37%)	109,076 (23.83%)	109,425 (25.89%)	108,408 (26.07%)	534,257 (23.61%)
Sex													
Men	1,111,050	0.88	0.77	0.85	0.90	0.97	0.95	42,684 (18.66%)	48,688 (20.38%)	48,501 (21.43%)	48,794 (23.24%)	46,950 (22.68%)	235,617 (21.21%)
Women	1,151,648	1.06	0.90	0.99	1.07	1.16	1.21	55,672 (22.22%)	60,304 (24.30%)	60,575 (26.19%)	60,631 (28.50%)	61,458 (29.43%)	298,640 (25.93%)
Age group													
00–04	95,713	0.11	0.10	0.10	0.11	0.13	0.15	144 (0.70%)	179 (0.81%)	189 (0.89%)	146 (1.17%)	275 (1.41%)	933 (0.97%)
05–11	139,135	0.17	0.13	0.16	0.17	0.18	0.21	352 (1.18%)	491 (1.54%)	513 (1.73%)	442 (1.88%)	577 (2.39%)	2,375 (1.71%)
12–17	104,694	0.17	0.13	0.16	0.17	0.18	0.20	395 (1.56%)	447 (1.90%)	400 (1.90%)	363 (2.08%)	412 (2.38%)	2,017 (1.93%)
18–34	449,216	0.26	0.20	0.25	0.27	0.28	0.31	2,952 (3.10%)	3,857 (4.02%)	4,076 (4.57%)	4,141 (4.90%)	4,609 (5.48%)	19,635 (4.37%)
35–44	388,117	0.44	0.35	0.41	0.45	0.46	0.51	5,032 (6.99%)	7,185 (8.70%)	7,633 (9.50%)	7,459 (9.91%)	8,675 (11.14%)	35,984 (9.27%)
45–54	352,274	0.76	0.64	0.74	0.78	0.80	0.89	12,093 (15.68%)	14,171 (18.33%)	13,932 (19.67%)	13,217 (20.27%)	14,143 (22.90%)	67,556 (19.18%)
55–64	280,851	1.40	1.17	1.35	1.44	1.50	1.60	20,737 (31.89%)	22,424 (36.50%)	22,374 (39.38%)	21,182 (40.60%)	19,678 (43.34%)	106,395 (37.88%)
65–69	119,582	2.00	1.74	1.95	2.07	2.07	2.21	12,363 (47.63%)	13,030 (53.38%)	13,364 (56.03%)	13,547 (56.26%)	12,571 (59.07%)	64,875 (54.25%)
70–74	90,096	2.43	2.16	2.36	2.51	2.52	2.62	10,263 (57.34%)	11,223 (62.45%)	11,566 (65.50%)	12,343 (65.88%)	12,046 (67.55%)	57,441 (63.76%)
75–79	97,240	2.91	2.62	2.81	2.97	2.99	3.12	12,213 (65.92%)	13,679 (70.14%)	13,917 (73.14%)	15,074 (73.41%)	14,792 (75.29%)	69,675 (71.65%)
80–84	75,474	3.26	2.97	3.22	3.34	3.31	3.46	10,950 (71.63%)	11,721 (75.99%)	11,456 (78.24%)	11,902 (77.48%)	11,644 (78.89%)	57,673 (76.41%)
85+	70,306	3.05	2.78	3.03	3.13	3.14	3.24	10,862 (66.40%)	10,585 (71.20%)	9,656 (71.76%)	9,609 (72.06%)	8,986 (73.12%)	49,698 (70.69%)

Note: Deprivation index is arranged in quintiles. The higher socioeconomic status is represented as 1, while the lowest one corresponds to 5).

**Table 2:** Healthcare spending by number of chronic conditions.

No. of chronic diseases	% of population	% of overall spending					
		Primary care	Specialized outpatient care	Emergency department attendances	Prescriptions	Inpatient stays	Total
0	57.08	29.22	23.09	44.58	4.84	13.29	19.43
1	19.31	20.8	20.61	19.69	13.98	12.97	17.02
2	10.04	15.63	16.54	10.43	18.53	13.29	15.26
3	5.82	11.65	12.73	7.2	18.28	12.94	13.15
4	3.41	8.25	9.23	5.37	14.97	11.59	10.5
5+	4.34	14.44	17.8	12.73	29.4	35.91	24.65
Total	100	100	100	100	100	100	100
1+ conditions (i.e chronic patients)	42.92	70.78	76.91	55.42	95.16	86.71	80.57
2+ conditions (i.e., multimorbid patients)	23.61	49.98	56.3	35.73	81.18	73.74	63.55

**Table 3:** Comparison of medians of annual primary care costs per patient, depending on number of chronic diseases and deprivation index.

PRIMARY CARE COST: median (percentile 25th–75th)													
No, Chronic conditions	Deprivation Index										*All*		
	1		2		3		4		5				
0	37 €	(0–150)	74 €	(0–189)	74 €	(0–204)	74 €	(0–202)	74 €	(0–215)	74 €	(0–189)	*
1–3	267 €	(137–447)	296 €	(150–479)	302 €	(152–491)	316 €	(165–506)	317 €	(167–509)	300 €	(150–486)	*
4–6	562 €	(360–845)	606 €	(401–892)	623 €	(412–903)	630 €	(418–911)	623 €	(417–899)	611 €	(402–892)	*
7–9	861 €	(558–1264)	898 €	(589–1318)	946 €	(634–1361)	924 €	(621–1301)	923 €	(632–1290)	912 €	(610–1304)	*
10+	1,225 €	(861–1803)	1,303 €	(883–1817)	1,252 €	(832–1818)	1,268 €	(913–1758)	1,283 €	(885–1774)	1,264 €	(872–1790)	#
***All***	***113 €***	***(0–313)***	***152 €***	***(37–365)***	***176 €***	***(37–387)***	***185 €***	***(37–406)***	***189 €***	***(37–417)***	***163 €***	***(37–376)***	*

^*^p<0.001;

# non-significant.

**Table 4:** Comparison of medians of annual specialised care costs per patient, depending on number of chronic diseases and deprivation index.

SPECIALISED CARE COST without hospitalization, but including A&E: median (percentile 25th–75th)													
No, Chronic conditions	Deprivation Index										*All*		
	1		2		3		4		5				
0	0 €	(0–153)	0 €	(0–164)	0 €	(0–164)	0 €	(0–164)	0 €	(0–164)	0 €	(0–164)	*
1–3	164 €	(0–410)	164 €	(0–484)	164 €	(0–492)	164 €	(0–492)	246 €	(0–563)	164 €	(0–492)	*
4–6	410 €	(153–891)	481 €	(164–973)	492 €	(164–1019)	552 €	(235–1044)	563 €	(246–1055)	492 €	(164–984)	*
7–9	727 €	(317–1372)	820 €	(399–1493)	820 €	(410–1535)	891 €	(410–1596)	891 €	(481–1607)	820 €	(410–1535)	*
10+	1,214 €	(634–2235)	1,225 €	(645–2077)	1,279 €	(705–2018)	1,307 €	(727–2144)	1,285 €	(705–2160)	1,278 €	(694–2122)	#
***All***	***0 €***	***(0–246)***	***82 €***	***(0–328)***	***82 €***	***(0–328)***	***82 €***	***(0–399)***	***153 €***	***(0–410)***	***82 €***	***(0–328)***	*

^*^p<0.001;

# non-significant.

**Table 5:** Comparison of medians of prescriptions costs per patient, depending on number of chronic diseases and deprivation index.

PRESCRIPTIONS: median (percentile 25th–75th)													
No, Chronic conditions	Deprivation Index										*All*		
	1		2		3		4		5				
0	0 €	(0–4)	0 €	(0–8)	0 €	(0–9)	0 €	(0–10)	0 €	(0–10)	0 €	(0–8)	*
1–3	70 €	(6–319)	66 €	(8–295)	65 €	(8–292)	70 €	(10–305)	65 €	(9–293)	67 €	(8–301)	*
4–6	626 €	(269–1183)	643 €	(283–1217)	645 €	(285–1209)	650 €	(287–1240)	646 €	(277–1231)	642 €	(281–1217)	*
7–9	1,118 €	(579–1863)	1,184 €	(611–1959)	1,202 €	(640–1985)	1,204 €	(654–1993)	1,186 €	(612–1978)	1,180 €	(623–1959)	*
10+	1,509 €	(778–2446)	1,639 €	(923–2528)	1,686 €	(933–2599)	1,627 €	(970–2588)	1,612 €	(944–2552)	1,612 €	(921–2557)	**
***All***	***2 €***	***(0–61)***	***6 €***	***(0–77)***	***8 €***	***(0–88)***	***10 €***	***(0–110)***	***10 €***	***(0–106)***	***7 €***	***(0–87)***	*

^*^p<0.001;

^**^p<0,05.

**Table 6:** Comparison of medians of hospitalizations costs per patient, depending on number of chronic diseases and deprivation index.

HOSPITALIZATIONS: percentage of citizens with no admissions(%); median (percentile 25th–75th) of costs of patients with any																			
No, Chronic conditions	Deprivation Index															*All*			
	1			2			3			4			5						
0	97%	2,075 €	(1602–3034)	97%	1,979 €	(1602–2732)	97%	1,979 €	(1602–2734)	97%	1,979 €	(1602–2831)	96%	1,979 €	(1602–2734)	97%	1,979 €	(1602–2858)	*
1–3	92%	3,046 €	(2164–5629)	91%	2,800 €	(2148–5092)	91%	2,841 €	(2129–4963)	91%	2,800 €	(2156–5106)	91%	2,734 €	(2071–4929)	91%	2,853 €	(2148–5152)	*
4–6	78%	4,692 €	(2532–9126)	76%	4,593 €	(2475–9042)	76%	4,632 €	(2502–9037)	75%	4,517 €	(2476–8949)	74%	4,420 €	(2436–8608)	76%	4,563 €	(2497–8977)	*
7–9	56%	5,944 €	(3425–11230)	53%	6,514 €	(3420–12417)	53%	6,442 €	(3420–12455)	52%	6,499 €	(3420–12620)	52%	6,202 €	(3420–11744)	53%	6,335 €	(3420–12132)	*
10+	30%	10,300 €	(4692–18340)	30%	8,747 €	(4556–16519)	29%	8,771 €	(4409–16609)	30%	8,839 €	(4385–15240)	32%	9,908 €	(4563–16814)	30%	9,378 €	(4495–16593)	#
***All***	94%	***3,014 €***	***(1996–5806)***	93%	***2,797 €***	***(1979–5693)***	93%	***2,897 €***	***(1979–5763)***	92%	***3,009 €***	***(2031–5864)***	92%	***2,936 €***	***(1992–5806)***	93%	***2,936 €***	***(1992–5806)***	*

^*^p<0.001;

# non-significant.

**Table 7:** Comparison of medians of total health care costs per patient, depending on number of chronic diseases and deprivation index.

TOTAL COST: median (percentile 25th–75th)													
No, Chronic conditions	Deprivation Index										*All*		
	1		2		3		4		5				
0	82 €	(0–324)	147 €	(0–405)	153 €	(0–426)	153 €	(0–424)	162 €	(0–465)	139 €	(0–404)	*
1–3	682 €	(320–1312)	724 €	(345–1397)	743 €	(356–1443)	780 €	(377–1498)	799 €	(384–1543)	744 €	(355–1437)	*
4–6	2,108 €	(1242–4100)	2,278 €	(1348–4433)	2,302 €	(1376–4545)	2,391 €	(1433–4709)	2,414 €	(1433–4753)	2,303 €	(1369–4535)	*
7–9	4,461 €	(2440–9341)	5,097 €	(2720–10450)	5,143 €	(2819–10549)	5,207 €	(2832–10701)	5,172 €	(2820–10450)	5,048 €	(2734–10349)	*
10+	10,275 €	(5210–20071)	10,280 €	(4960–19189)	10,331 €	(5341–18544)	10,178 €	(5270–18380)	9,956 €	(5011–18653)	10,212 €	(5130–18767)	#
***All***	***261 €***	***(26–798)***	***342 €***	***(68–939)***	***373 €***	***(77–1011)***	***402 €***	***(82–1096)***	***431 €***	***(93–1149)***	***356 €***	***(65–988)***	*

^*^p<0.001;

# non-significant.

We use the Generalized Linear Model (GLM) with gamma distribution and log link [29] to evaluate the relationship between the number of chronic conditions and cost after adjusting for confounding factors. As Healthcare cost data are typically non-normally distributed with a skew towards the right, Gamma regression is a better modeling approach to deal with this skewness than Ordinary Least Square (OLS) regression [30], [31], [32]). In our model, individual total cost was our dependent variable. The independent variables we use were sex, age (by groups), DI, and the number of chronic conditions. Given that age groups behave differently of males and females in terms of the utilization of health services and its cost, we also allowed for the interaction of age and sex. Predicted mean adjusted costs were obtained by using the recycled predictions methodology [33], [34]. This method calculates the mean adjusted cost for each category in the variable of interest as the average of all individual predictions based on the regression model when all subjects are assigned to such category, while holding constant all other model covariates.

Given that our goal is to observe the relationship between the increase in individual cost and the number of chronic conditions, we chose, by consensus within the research team, 10 among the most common pathologies and whose cost is more significant. At the same time, our methodology allows us to show how the increase in individual cost is related to the characteristics of specific pathologies. Hence, we run a log-gamma model separately for each pathology using as independent variables age, sex, DI, the number of other pathologies suffered by the patient, and the interaction of each pathology with the number of other pathologies. Out of those estimation models, we obtain the mean adjusted cost (by recycled predictions methods) and analyze whether they vary when the number of comorbidities increases.

All this analysis was performed using SAS (version 9.2).

## Results

### Prevalence of Multimorbidity

Table 1 shows the distribution of the population by age group as well as the average number of chronic diseases per patient, this being 0.97 overall. The percentage of the population with multimorbidity is 23.61%, representing 55% of patients with any chronic condition, given that 42.92% have at least one chronic condition (as shown in Table 2).

Chronic conditions are more common in females than in males, and their prevalence rises with age up to 85 years old, when there is a slight decrease. Multimorbidity affects more than half of the population over 65 and more than three quarters of those between 80 and 84 years old.

### Multimorbidity and Socioeconomic Status

Those with a greater number of illnesses tended to live in more deprived areas. This pattern is seen in both sexes and all age groups. When comparing the most and least deprived groups, the differences are larger, in relative terms, at younger ages and somewhat less so in the older groups (Table 1).

### Concentration of Health Expenditures in Multimorbid Patients

Table 2 shows the percentage of healthcare spending by number of chronic diseases. Chronic patients (42.92% of the population) are responsible for 80.57% of total healthcare expenditures (86.71% for inpatient care and 95.16% for prescriptions). Importantly, even in the case of A&E, spending on chronic patients represents more than half of the total (55.42%). The concentration of health expenditures is evidenced by the fact that 23.61% of patients have multiple conditions (multimorbidity according to the definition used for this study) but they account for 63.55% of total healthcare expenditures. Of those, patients with five of more chronic conditions, representing less than 5% of the population (4.33%), consume almost a quarter of the total healthcare resources (24.65%).

### Health Expenditures and Socioeconomic Status

The average cost of the healthcare provided for each patient was €1,124. Tables 3–7 show the median, 25^th^ and 75^th^ percentile of costs as a function of number of chronic conditions and DI. According to these indicators, the total healthcare cost per person is slightly higher in more deprived social areas. The same trend is seen comparing the figures of the groups of patients with different numbers of chronic diseases. Those differences are statistically significant (p<0.001) for all different categories of health expenditure, both for the general population and for each specific group of multimorbid patients, but for those individuals with 10 or more pathologies, where only the difference in expenditures in pharmaceutical prescriptions is statistically significant (p<0.05).

### Regression Analisis: Relationships between Health Expenditures and Number of Chronic Diseases

We present the results from the Generalized Linear Model (GLM) with gamma distribution in the supporting material (table B in File S2). From those estimations we have obtained the average individual cost adjusted by the independent variables age, sex DI and number of pathologies, as shown in tables 8 and 9.

**Table 8:** Healthcare expenditure.

Age Group		Male	Female
	00–04	2,550	2,316
	05–11	1,125	1,057
	12–17	933	859
	18–34	798	1,298
	35–44	818	1,300
	45–54	921	1,033
	55–64	1,098	1,083
	65–69	1,231	1,156
	70–74	1,262	1,187
	75–79	1,303	1,209
	80–84	1,254	1,161
	85+	1,078	1,030

Means, adjusted by generalized linear regression model, of several groups of population.

**Table 9:** Healthcare expenditure.

Deprivation Index	1	976
	2	1,117
	5	1,155
	4	1,186
	5	1,226
Number of Chronic Diseases	0	380
	1	1,017
	2	1,735
	3	2,533
	4	3,406
	5	4,506
	6	5,799
	7	7,348
	8	8,759
	9	10,417
	10+	13,891

Means, adjusted by generalized linear regression model, of several groups of population.

Once adjusted per number of chronic conditions and socioeconomic characteristics, the effect of ageing is small. Furthermore, individual cost for children (with an equal number of diseases) is greater than for other ranges of age. With respect to age, individual cost for females is lower than for males for all ages but in the range of 18–44 years old, as expected due to obstetric care.

We also analyze the effect of socioeconomic status through the DI. The average individual cost for those in the most deprived socioeconomic group is significantly (25%) higher than that of individuals in the most favorable socioeconomic group.

The increase in healthcare expenditures observed in the number of chronic diseases is shown in Table 9. The annual average cost for patients with one chronic disease is €637 higher than that for people with none, while the effect of “adding” a second, third or fourth disease becomes progressively more expensive and, for example, the cost of another illness after the eighth rises to an additional € 1,657 per year.

### Health Expenditures for Patients with Specific Conditions

**Figure 1:**
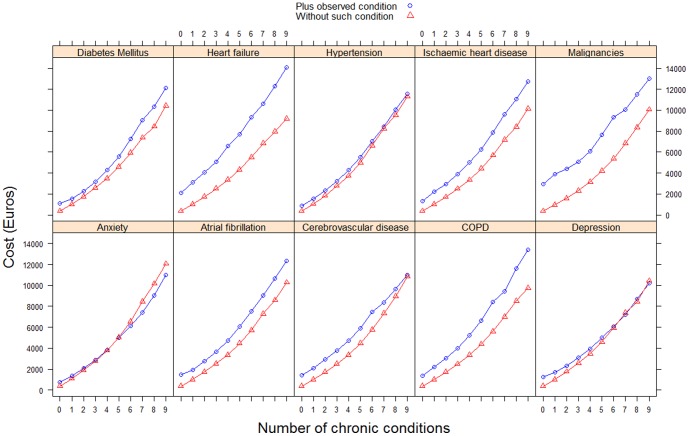
Difference in average of adjusted annual cost (in euros) per patient, by number of coexisting chronic diseases according to the presence of certain selected diseases.

In our analysis, we find that it is not only the number of chronic diseases what matters when looking at the increase in cost, but also which are the conditions suffered by patients and specifically, which is the considered primary condition. Table 10 and figure 1 present average costs by coexistence of other conditions for some specific health conditions. On the one hand, for chronic obstructive pulmonary disease (COPD), diabetes mellitus, ischaemic heart disease and heart failure there are progressive increases according to the number of other coexisting conditions (there being a two- to three-fold difference between the annual cost of patients with and without any of these four conditions, in the case of patients with another eight conditions). On the other hand, the reverse is true in the cases of depression or anxiety, greater comorbidity leading to progressively smaller rises and even negative differences for comorbidity of seven or more chronic diseases, and malignancies or cerebrovascular disease, with costs rising up to a point (other six diseases) and falling afterwards.

**Table 10:** Difference in average of adjusted annual cost (in euros) per patient, by number of coexisting chronic diseases according to the presence of certain selected diseases.

	Anxiety			Atrial Fibrilation			Cerebrovascular disease			COPD			Depression		
N Chronic Conditions	w/o such condition	+Observed condition	Difference	w/o such condition	+Observed condition	Difference	w/o such condition	+Observed condition	Difference	w/o such condition	+Observed condition	Difference	w/o such condition	+Observed condition	Difference
**0**	375	742	**366**	379	1,504	**1,125**	379	1,395	**1,015**	379	1,357	**978**	379	1,254	**875**
**1**	1,098	1,344	**246**	1,015	1,915	**900**	1,014	2,080	**1,066**	1,012	2,191	**1,179**	1,013	1,694	**680**
**2**	1,906	2,102	**196**	1,732	2,749	**1,017**	1,727	2,948	**1,221**	1,720	3,031	**1,311**	1,739	2,324	**585**
**3**	2,777	2,850	**72**	2,522	3,652	**1,129**	2,510	3,770	**1,260**	2,502	4,014	**1,512**	2,566	3,087	**521**
**4**	3,780	3,841	**61**	3,381	4,741	**1,361**	3,369	4,751	**1,381**	3,343	5,236	**1,893**	3,482	3,914	**432**
**5**	5,052	5,000	**−52**	4,467	6,091	**1,624**	4,472	5,898	**1,426**	4,397	6,621	**2,224**	4,701	4,993	**292**
**6**	6,544	6,135	**−409**	5,718	7,526	**1,808**	5,788	7,484	**1,696**	5,623	8,422	**2,799**	6,131	6,051	**−80**
**7**	8,473	7,417	**−1,056**	7,293	9,052	**1,759**	7,324	8,385	**1,061**	7,023	9,422	**2,399**	7,962	7,183	**−779**
**8**	10,167	9,012	**−1,154**	8,599	10,643	**2,045**	8,943	9,646	**704**	8,523	11,598	**3,075**	9,658	8,717	**−941**
**9**	12,077	11,000	**−1,077**	10,278	12,347	**2,070**	10,855	11,003	**149**	9,765	13,399	**3,635**	11,484	10,231	**−1,253**

	Diabetes Mellitus			Heart failure			Hypertension			Ischaemic heart disease			Malignancies		
	w/o such condition	+Observed condition	Difference	w/o such condition	+Observed condition	Difference	w/o such condition	+Observed condition	Difference	w/o such condition	+Observed condition	Difference	w/o such condition	+Observed condition	Difference
**0**	380	1,101	**721**	379	2,123	**1,744**	383	868	**485**	379	1,296	**916**	379	2,964	**2,585**
**1**	1,014	1,532	**518**	1,016	3,108	**2,091**	1,055	1,573	**518**	1,016	2,236	**1,220**	961	3,907	**2,945**
**2**	1,763	2,289	**526**	1,733	4,044	**2,311**	1,851	2,336	**484**	1,720	2,934	**1,214**	1,593	4,403	**2,811**
**3**	2,596	3,192	**595**	2,519	5,081	**2,562**	2,785	3,206	**421**	2,505	3,911	**1,406**	2,333	5,077	**2,744**
**4**	3,484	4,288	**804**	3,354	6,600	**3,245**	3,739	4,273	**534**	3,348	5,025	**1,676**	3,153	6,064	**2,911**
**5**	4,607	5,551	**944**	4,342	7,716	**3,374**	4,993	5,504	**512**	4,424	6,238	**1,814**	4,209	7,654	**3,445**
**6**	5,936	7,278	**1,342**	5,518	9,326	**3,808**	6,635	7,035	**400**	5,704	7,862	**2,159**	5,372	9,350	**3,978**
**7**	7,379	9,070	**1,690**	6,827	10,601	**3,775**	8,246	8,430	**184**	7,201	9,610	**2,409**	6,834	10,069	**3,235**
**8**	8,439	10,348	**1,909**	7,947	12,317	**4,370**	9,540	10,033	**494**	8,434	11,040	**2,605**	8,377	11,510	**3,133**
**9**	10,448	12,133	**1,684**	9,199	14,069	**4,869**	11,302	11,564	**262**	10,140	12,738	**2,598**	10,073	13,000	**2,927**

Note: These series have been truncated, because patients with +10 chronic diseases are very scarce (<0.01% of total population); beyond such limit, the groups exhibit a great variability in their results due to their small size.

## Discussion

In this paper, we utilize a unique database on the entire population of the Basque Country. The Basque Country being a region in Spain, its size in terms of population, more than two million people, is similar to that from small countries in Europe like Lithuania, Slovenia or Latvia, and only half of the population than countries as Norway, Ireland or Croatia, being greater than almost half of the States in the US. The uniqueness of our data set -with respect to related articles in the literature-, consists in it containing four years of individual -clinical and cost- data from different sources of information to cover a great proportion of the public resources used, for the entire population of the Basque Country, as well as an indicator of socioeconomic status. Hence, we avoid the potential biases of taking into account only a proportion of the population (such as the elderly), one type of healthcare service provision (such as primary care), or using information only from some selected health centres.

We show evidence of how multimorbidity is a common phenomenon and its presence increases with the age of the population considered, and of the concentration of healthcare expenditures in patients with multimorbidity). Together with the process of ageing in the population, common to all developed countries, our results add to the body of evidence justifying the great international concern about public health provision, planning, and funding for patients with multimorbidity in the near future. Furthermore, there are two respects in which the Basque Country is ahead of other regions and countries in the need for efficiency in health provision, making these findings particularly relevant for the international audience. First, the population in the Basque Country is already slightly more aged than that of Spain as a whole [35], one of the most aged developed countries. Second, the current economic conditions in Spain produces a sense of urgency among policy makers and is resulting in numerous implementations of policies promoting direct and indirect cuts in health expenditures [36].

An innovative pillar of our analysis is to include a socioeconomic indicator and explore its relationship with the prevalence and healthcare expenditures of patients with multimorbidity. We show evidence that both the prevalence of multimorbidity and thus the level of need in provision, are greater in more deprived geographical areas. In the Basque Country, there is no evidence of inequity (or discrimination against populations in more deprived areas) as the greater the need, the greater the level of public health expenditures. This reflects decades of planning and development of primary care services in the National Health Service. However, the greater prevalence of multimorbid patients in more deprived areas is of public concern in itself and should be taken into account as a gradient between health and wealth.

We also find evidence of how, on average, the annual use of healthcare resources by chronically ill patients grows as the number of chronic diseases increases. Notably, our data suggests that this increase is not linear but rather tends to be progressive. This is the most common pattern and, it would seem, the most expected for the prevalence of all chronic conditions. There are exceptions, however, namely depression and anxiety: patients with either of these conditions and more than five (in the case of depression) or two (in the case of anxiety) other chronic diseases, use a lower level of resources than if they only had these other chronic health problems. In other conditions, such as malignant neoplasms or cerebrovascular disease, healthcare costs increase progressively with multimorbidity, the magnitude of the increase growing for up to six more diseases but falling thereafter. That result, although initially paradoxical, might be explained through different interpretations. Patients with some of those chronic conditions (as mental condition) might be reluctant to look for or might find more obstacles to access health service provision than individuals without those conditions. Also, clinical records for patients with multiple diseases and the elderly can be particularly difficult [37] and therefore, their diagnoses are less accurate. Hence, physicians may not record a diagnosis of anxiety or depression when there seems to be a clear reason for it. At the same time, it is also plausible that in the case of some especially severe conditions, as malignancies, treatments tend to be more aggressive for patients with less comorbidities than in those, more complex patients, with a greater combination of chronic problems. Lastly, although we do account for the number of chronic diseases and which conditions are those, we have no information on their level of severity, and for each specific case, some might be more severe than others.

We have shown how in the Basque Country health expenditures are concentrated in patients with multimorbidity, and within this group, the most expensive are those with heart failure, ischaemic heart disease, and diabetes mellitus among other conditions. Having identified the most expensive populations in terms of public health expenditures, the next step is to design strategies and undertake proactive interventions specifically for those patients. The aforementioned “Strategy for tackling the challenge of chronicity in the Basque Country” [19], launched in 2010, already encouraged changes in this direction. Our most recent results, however, underline the need to keep making further efforts in the re-organisation of health provision specifically for the patients identified, who are those that will benefit the most from increasing care coordination. In this area, integrated care approaches for patients with multiple conditions are increasingly popular in Spain and preliminary evaluations seem promising in terms of their effectiveness [38].

The goal is to improve the level of control of the relevant indicators for patients identified as being most in-need or at-risk, e.g., those that are most expensive, are readmitted most frequently or fulfil various clinical criteria [39], [40].

Our study has certain limitations. Firstly, administrative databases obviously only contain information about health problems for which people seek medical care. Therefore, the prevalence of diseases only reflects attended morbidity excluding conditions that might be present but have not yet manifested or have not been detected by either the patients or their doctors. This is quite common in chronic diseases and it is influenced by various factors, such as accessibility to healthcare services, though this is not questioned in our setting for the case of the Basque Health Service. Secondly, our database does not contain information on psychiatric hospitals; and, even though patients admitted in such hospitals are also usually cared for by primary care doctors, given their special characteristics, it is possible that their health records were not as complete as for the rest of the population. Thirdly, employing socioeconomic indicators at the level of area of residence, our study has the limitations common to ecological studies. Finally, cost data are restricted to expenditures funded by the public sector, and individual health care costs were calculated from the standard pricing of the services provided, with the exception of drug costs (based on their market value) and DRGs (average costs from cost accounting). That is, certain costs have not been considered, namely the costs of activities not included in the dataset, e.g., admissions to mental health hospitals, home hospitalisation, etc. (we estimate that 21.58% of the total cost is not included in our analysis). We interpret the lack of data on use and costs in private health centres as a plausible explanation for the greater need (and higher demand) for outpatient services from more deprived socioeconomic areas. However, even if that were to be true, the fact that hospitalisation is similar for all socioeconomic groups demonstrates the trust of the entire population in the public provision under the national health service for expensive treatments and maybe some adverse selection in the private health insurance market. Further, this paper is focused on the organisation of public health service provision and planning, and thus, private health provision is beyond the scope of our analysis. At the same time, the fact that some health expenditures are derived from cost standardisation might explain the lower concentration of health expenditures in the most expensive patients compared to other studies in the literature, given that the level of prices (unit cost) is in Spain lower than in that in other countries.

In essence, multimorbidity occurs as a complex phenomenon and patients with multiple health problems do not share a common set of characteristics. It is known that the relationship between comorbidity, quality and healthcare outcomes is not uniform, and depends upon the specific combination of diseases [3]. Future studies are needed to determine whether the observed higher expenditures among multimobid patients are justified or not, in order to detect possible inefficiencies in their care. Besides, further research is required to characterize the diverse subgroups of patients with multimorbidity, to implement specific, patient-centred care programmes.

## Conclusions

Multimorbidity is a very common finding, its prevalence rises with age, and it is related to unfavourable socioeconomic factors. The costs of caring for chronic patients tend to increase dramatically with the number and combination of comorbidities, although the pattern varies for certain specific diseases. Our paper using comprehensive data on the utilization of all levels of public health services by the entire population of the Basque Country shows the burden of multimorbidity in a population engaged in an irreversible process of population ageing and the implications of multimorbidity for patients. Moreover, the current economic crisis has pushed the Basque Country, in search of efficiency, to innovate in the study of multimorbidity and its effect in patients, families and caregivers. This is perhaps the most important current challenge for policy makers, administrators, clinicians, and researchers in our health system, and probably also in that of other developed countries, to guarantee the quality of care provided.

## Supporting Information

File S1 **Definition of Chronic Conditions.**
(DOCX)

File S2 **Generalized linear model (GLM) with gamma distribution.**
(DOCX)
