# Supplementary material for: Prevalence and Costs of Multimorbidity by Deprivation Levels in the Basque Country: A Population Based Study Using Health Administrative Databases
Source: PLoS One. 2014 Feb 27;9(2):e89787. doi: 10.1371/journal.pone.0089787 (PMC3937325; doi:10.1371/journal.pone.0089787)
Supplement: File S2 — Generalized linear model (GLM) with gamma distribution. (DOCX) [file pone.0089787.s002.docx]

File S2. Supporting Information

Table B: Generalized linear model (GLM) with gamma distribution.

| **Variable  (Reference)** | **Estimate** | **Standard  Errors** | **Wald 95%  Confidence interval Lower - Higher** | | **Wald  Chi-square** | **Pr > ChiSq** |
| --- | --- | --- | --- | --- | --- | --- |
| **Sex (Female)** | |  |  |  |  |  |
| Male | 0.0458 | 0.0082 | 0.0298 | 0.0619 | 31.31 | <.0001 |
| **Deprivation Index (Hightest level 5)** | | |  |  |  |  |
| Level 1 | -0.2282 | 0.0021 | -0.2324 | -0.224 | 11539.4 | <.0001 |
| Level 2 | -0.0925 | 0.0021 | -0.0966 | -0.0883 | 1914.8 | <.0001 |
| Level 3 | -0.0593 | 0.0021 | -0.0635 | -0.0551 | 766.78 | <.0001 |
| Level 4 | -0.0328 | 0.0022 | -0.037 | -0.0285 | 224.6 | <.0001 |
| **Age band (85+)** | |  |  |  |  |  |
| 00-04 | 0.8102 | 0.0067 | 0.7972 | 0.8233 | 14742.4 | <.0001 |
| 05-11 | 0.0258 | 0.0062 | 0.0137 | 0.0379 | 17.57 | <.0001 |
| 12-17 | -0.1815 | 0.0065 | -0.1943 | -0.1687 | 773.1 | <.0001 |
| 18-34 | 0.2313 | 0.0052 | 0.2211 | 0.2416 | 1962.53 | <.0001 |
| 35-44 | 0.2327 | 0.0053 | 0.2223 | 0.243 | 1952.45 | <.0001 |
| 45-54 | 0.0031 | 0.0052 | -0.0072 | 0.0134 | 0.35 | 0.5531 |
| 55-64 | 0.0498 | 0.0053 | 0.0394 | 0.0603 | 87.77 | <.0001 |
| 65-69 | 0.1158 | 0.0061 | 0.1039 | 0.1277 | 362.66 | <.0001 |
| 70-74 | 0.1419 | 0.0064 | 0.1293 | 0.1545 | 487.19 | <.0001 |
| 75-79 | 0.1601 | 0.0062 | 0.1479 | 0.1722 | 662.52 | <.0001 |
| 80-84 | 0.1195 | 0.0065 | 0.1068 | 0.1323 | 335.9 | <.0001 |
| **Number of chronic conditions (0)** | | |  |  |  |  |
| 1 | 0.985 | 0.0018 | 0.9814 | 0.9886 | 290633 | <.0001 |
| 2 | 1.5197 | 0.0025 | 1.5149 | 1.5246 | 379493 | <.0001 |
| 3 | 1.898 | 0.0031 | 1.8918 | 1.9042 | 365480 | <.0001 |
| 4 | 2.194 | 0.004 | 2.1863 | 2.2018 | 306294 | <.0001 |
| 5 | 2.474 | 0.0051 | 2.464 | 2.4839 | 238355 | <.0001 |
| 6 | 2.7262 | 0.0066 | 2.7132 | 2.7393 | 168520 | <.0001 |
| 7 | 2.963 | 0.0088 | 2.9458 | 2.9802 | 114071 | <.0001 |
| 8 | 3.1387 | 0.0118 | 3.1156 | 3.1618 | 71037.4 | <.0001 |
| 9 | 3.312 | 0.016 | 3.2807 | 3.3433 | 42989.2 | <.0001 |
| 10+ | 3.5998 | 0.0159 | 3.5687 | 3.631 | 51275.7 | <.0001 |
| **Interaction sex and age band (85+ female)** | | | |  |  |  |
| 00-04 Male | 0.0505 | 0.0104 | 0.0301 | 0.071 | 23.43 | <.0001 |
| 05-11 Male | 0.0163 | 0.0098 | -0.0029 | 0.0354 | 2.76 | 0.0968 |
| 12-17 Male | 0.037 | 0.0103 | 0.0169 | 0.0571 | 12.98 | 0.0003 |
| 18-34 Male | -0.5324 | 0.0087 | -0.5495 | -0.5154 | 3732.45 | <.0001 |
| 35-44 Male | -0.5093 | 0.0088 | -0.5265 | -0.4921 | 3353.16 | <.0001 |
| 45-54 Male | -0.1613 | 0.0089 | -0.1786 | -0.1439 | 331.92 | <.0001 |
| 55-64 Male | -0.0313 | 0.009 | -0.049 | -0.0136 | 12.07 | 0.0005 |
| 65-69 Male | 0.0164 | 0.01 | -0.0033 | 0.036 | 2.67 | 0.1025 |
| 70-74 Male | 0.0155 | 0.0106 | -0.0053 | 0.0362 | 2.14 | 0.1436 |
| 75-79 Male | 0.0293 | 0.0104 | 0.0088 | 0.0497 | 7.86 | 0.005 |
| 80-84 Male | 0.0315 | 0.0111 | 0.0098 | 0.0532 | 8.12 | 0.0044 |
